# Tuberculin Treatment in General Practice

**Published:** 1908-07-25

**Authors:** J. E. Gordon

**Affiliations:** Assistant Resident Medical Officer, Royal National Hospital for Consumption, Ventnor, Isle of Wight.


### THE GENERAL PRACTITIONER'S COLUMN.

[Contributions to this Column are invited, and if accepted will be paid for.]

### TUBERCULIN TREATMENT IN GENERAL PRACTICE.

By J. E. GORDON, M.B. (Edin.), Assistant Resident Medical Officer, Royal National Hospital for Consumption, Ventnor, Isle of Wight.

THE association of tuberculin with the opsonic index treatment—and its supposed dependence thereon—is in many instances the reason which prevents general practitioners from using the latter. They appear to regard the estimation of the index an essential preliminary and accompaniment. Without practical knowledge of opsonic methods, they are tempted to leave aside the administration of a vaccine controlled by so complicated and laborious machinery.

It should be clearly understood that in many instances excellent results can be obtained from tuberculin without opsonic estimation. Thanks to the researches of Wright, the use of Koch's T.R. in localised lesions, as glands, ulcers, etc., has been placed on a simple footing, which every practitioner may appreciate. I refer to the exhibition of $\frac{1}{1000}$ mg T.R. at ten to fourteen days' interval or longer. In a suitable case, say a tuberculous joint, which has resisted ordinary treatment—granted there are no complications—the improvement after a few such injections is frequently astonishing.

I have treated many cases of the sort upon this plan, *e.g.* a large discharging tuberculous ulcer of the lumbar region of the back, six inches in diameter each way, probably originating as an abscess of bone. It healed after five injections of $\frac{1}{1000}$ mg. at fourteen days' interval. The ulcer had remained stationary for over six months.

An extensive lupus, involving the cheek of one side and the nose, was scraped, and a few days later treated with tuberculin. In ten weeks, during which time five injections of $\frac{1}{1000}$ mg. Koch's T.R. were given, the healing process was complete. This was a case which had resisted treatment for over a year.

I once treated a case of persistent pulmonary hæmorrhage with Koch's T.R., at three weeks' intervals between successive doses. The hæmoptysis appreciably diminished under three injections. Six hours after the fourth injection the patient became breathless and cyanosed, and was apparently dying. Previous to the injection, he had been quite well, or as well as could be expected in the condition. The alarming change which occurred was, of course, attributed by all to the injection. However, the true cause showed itself when the patient coughed up a cup-full of old blood clot, which immediately relieved his embarrassment. This occurred in a consumption hospital in the Midlands, where tuberculin had only been used spasmodically. Had the patient died, it would have been a difficult matter to convince critics that the death was not due to the treatment.

While the practitioner may find that a certain proportion of pulmonary tuberculosis benefits from tuberculin, he will be wise if he leave its administration in phthisis to the expert, at least at the commencement. The subject of tuberculin in pulmonary tuberculosis is a complicated one. Tuberculin gives best results as a supplement to ordinary sanatorium treatment, and cases require careful selection and consideration. An oscillating opsonic value, pyrexia and mixed infection are contra-indications. In such cases, the investigation of the opsonic index is advisable. Chronic apyrexial cases can be injected safely without estimation of the index.

Routine injection of all cases, good, indifferent, and bad, has been suggested on the ground that experience has shown the most hopeless may exhibit marked improvement under tuberculin. I would protest, however, against the injection of every patient with pulmonary phthisis. The percentage of successes is then submerged by the failures.

It is, however, in the department of surgical tuberculosis, where the lesions are localised in bone, joints, skin, glands, and such organs as the throat and bladder, that the practitioner will achieve success in the judicious use of tuberculin. Many persons in apparently good health carry a tuberculous focus of this nature, that may resist ordinary surgical interference and local applications. By raising the low opsonic index, which these cases exhibit, to and above the normal, tuberculin will produce results of a most gratifying character. The curette or knife may be necessary as a preliminary to evacuate pus, caseation, and other debris. Mixed infections should at the same time be treated. Tuberculous lesions complicated by sepsis may be found to heal remarkably well under tuberculin and a simple dressing, if drainage is good. Joint disease may require a splint to rest the part. An injection, previous to operative measures, may be indicated to minimise the risk of dissemination of bacilli and toxin into the system.

*Dosage.*—Koch's T.R. can be readily obtained through the ordinary chemist. It is dispensed in phials, each stated to contain $\frac{1}{1000}$ mg. (.001 mg.) of solid substance dissolved in fluid. These are sterile, and ready for injection. A few doses of $\frac{1}{1000}$ mg. may effect a cure in suitable cases.

As long as the disease is clearing up under the initial dose, there is no necessity to increase the dose. If there is a tendency for the healing process to slacken, the dose may be increased. The intervals between successive doses should be about a fortnight or three weeks. Children may be given $\frac{1}{2000}$ mg. to start with, increasing to $\frac{1\frac{1}{2}}{1000}$ mg. They will usually be found to take the latter straight away without any constitutional disturbance.

The initial dose in acute pulmonary tuberculosis, where the index shows a tendency to swing, should be less—say, $\frac{1}{10000}$ mg. to $\frac{1}{5000}$ mg. Increase is guided by the symptoms and opsonic reading.

On completion of the cure, injections should be continued at, say, monthly intervals, for at least a year, to avoid a return of the disease. Any recrudescence after cessation of treatment is an indication for its re-establishment.

*Modus Operandi.*—The needle of the hypodermic syringe is boiled, and boiling water drawn through the syringe itself. The syringe should then be laid aside to cool. No antiseptic should be used, or, if used, it should be washed away with sterile water. The site of injection may be cleaned with soap and water, followed by ether. The phial is broken at the scratch mark on the neck, and the fluid drawn into the syringe. Air is expelled, and the tuberculin injected directly under the skin, the needle being pushed in to its full extent.

The puncture requires no dressing. It is a good plan to inject as close to the disease as possible; for example, in a phthisis laryngea, under the skin in the middle line of the neck directly above the thyroid cartilage.

The general practitioner who wishes to be successful in the administration, therapeutically, of tuberculin, should pay attention to:

1. The careful selection of cases.
2. The admitted value of the small dose at long intervals.
3. The precautions already indicated in the actual mode of injection.

